# High-Altitude Cerebral Edema in a Non-climber at the K2 Base Camp: A Case Report

**DOI:** 10.7759/cureus.99151

**Published:** 2025-12-13

**Authors:** Muhammad U Faisal

**Affiliations:** 1 Emergency Medicine, University Hospitals Birmingham NHS Foundation Trust, Birmingham, GBR

**Keywords:** acetazolamide, altitude illness, ataxia, dexamethasone, expedition health, high altitude cerebral edema, hypoxia, k2 base camp, mountain medicine, wilderness medicine

## Abstract

A 47-year-old male base camp team member at the K2 Base Camp (5150 m) developed headache, dizziness, unsteady gait, intermittent diplopia, and mild confusion a few days after arrival. His vital signs were respiratory rate 20 breaths per minute, heart rate 112 beats per minute, blood pressure 130/85 mmHg, and oxygen saturation 87% on room air. Neurological examination revealed truncal ataxia and disorientation without focal deficits. In the resource-limited expedition setting, a clinical diagnosis of high-altitude cerebral edema (HACE) was made. The patient was treated with high-flow oxygen and dexamethasone 8 mg intramuscularly, followed by 4 mg every six hours (administered orally during descent), along with acetazolamide 250 mg twice daily and hydration. Helicopter evacuation was impossible for several days due to poor weather, and as the only doctor on site, the clinician arranged descent by mule with accompanying team members. Two days later, communication from a lower altitude confirmed full recovery. This case describes the presentation and successful field management of HACE in a non-climber working at an extreme altitude.

## Introduction

High-altitude cerebral edema (HACE) is a severe form of altitude-related illness associated with exposure to high elevations [1,2]. It is thought to involve hypobaric hypoxia leading to vasogenic cerebral edema [3]. Although typically reported among climbers, individuals working in support roles at altitude may also develop symptoms, particularly when acclimatization is inadequate [4,5]. Support staff at major expedition base camps may be exposed to risk due to rapid ascent schedules and physically demanding working conditions. This report describes the presentation and field management of a patient who became symptomatic at the K2 Base Camp, Pakistan, under resource-limited conditions.

## Case presentation

A 47-year-old male support team member at the K2 Base Camp (5150 m) developed a headache and dizziness in the morning after being at this altitude for a few days. Over the course of the day, his symptoms progressed to unsteady gait, visual blurring with intermittent diplopia, and increasing confusion by late afternoon. He had previously worked at high altitude without illness.

He was assessed the same day in the expedition medical tent. On examination, he was alert but disoriented. His vital signs were heart rate 112 bpm, blood pressure 130/85 mm Hg, respiratory rate 20 breaths per minute, and oxygen saturation 87% on room air, findings consistent with hypobaric hypoxia at this altitude. Although his respiratory rate was normal at rest, he became tachypneic with exertion, a physiologically expected response at 5150 meters. Neurological assessment demonstrated truncal ataxia and horizontal diplopia, with no focal motor or sensory deficits.

Based on the high-altitude setting, symptom progression, and absence of focal neurological signs, a clinical diagnosis of HACE was made [1]. Management included high-flow supplemental oxygen and dexamethasone 8 mg intramuscularly, followed by 4 mg every six hours (oral doses provided for continuation during descent). Acetazolamide 250 mg twice daily was provided, and hydration was encouraged.

Helicopter evacuation was not possible for several days due to adverse weather conditions. As the sole clinician at the base camp, descent was arranged by mule with accompanying team members. Two days later, communication from a lower altitude (~3000 m) confirmed complete recovery following descent. Table 1 summarizes the timeline of this case.

**Table 1:** Timeline of symptom progression, clinical findings, and management HR: heart rate; BP: blood pressure; RR: respiratory rate; IM: intramuscularly

Time/Stage	Events
Arrival at 5150 m (few days)	Working as support staff, previously well
Morning (day of presentation)	Headache, dizziness
Afternoon	Ataxia, diplopia, and increasing confusion
Assessment in the medical tent	HR 112 bpm, BP 130/85 mm Hg, RR 20 (At rest), SpO₂ 87%, truncal ataxia, horizontal diplopia
Initial management	High-flow oxygen, dexamethasone 8 mg IM → 4 mg every 6 hours, acetazolamide, hydration
Evacuation	Helicopter unavailable due to weather; descent by mule
Outcome	Full recovery at ~3000 m after 2 days

## Discussion

HACE is a medical emergency that can rapidly progress to coma or death if unrecognized [1,2]. It represents the severe end of the acute mountain sickness spectrum, resulting from hypobaric hypoxia and vasogenic cerebral edema [3]. Early neurological signs such as ataxia, confusion, and visual disturbance should trigger immediate treatment, as delays can be fatal.

The diagnosis of HACE is primarily clinical, especially in field or expedition environments where imaging or laboratory tests are unavailable [1,3]. Prompt administration of oxygen and dexamethasone, followed by descent, forms the cornerstone of management [4,5]. Current Wilderness Medical Society guidelines recommend an initial dexamethasone dose of 8 mg, followed by 4 mg every six hours, with acetazolamide (125-250 mg twice daily) as an adjunct to support acclimatization [5]. At altitudes above 5000 m, oxygen saturations in the 80s may occur even with a normal resting respiratory rate, which helps contextualize the patient’s initial vital signs [6,7].

This case emphasizes that individuals in non-climbing roles at altitude, such as porters and support staff, are equally at risk, particularly when ascent is rapid or acclimatization schedules are not followed [2,5]. Previous reports have also described HACE among non-climbers at high-altitude expedition camps, supporting the importance of recognizing risk beyond climbing personnel. It also demonstrates that timely intervention using available resources, such as oxygen, corticosteroids, and descent, can lead to full recovery even in austere, resource-limited settings.

## Conclusions

This case describes the development of neurological symptoms consistent with HACE in a non-climber working at the K2 Base Camp. The patient’s rapid assessment and timely management at base camp, followed by descent, were associated with a full recovery despite the remote and resource limited environment. This report highlights the practical challenges and successful field management of an unwell team member at extreme altitude, while acknowledging that the diagnosis was made clinically in a setting where imaging and formal investigations were not available.
